# Factors associated with postprandial lipemia and apolipoprotein A-V levels in individuals with familial combined hyperlipidemia

**DOI:** 10.1186/1472-6823-14-90

**Published:** 2014-11-25

**Authors:** Paloma Almeda-Valdes, Daniel Cuevas-Ramos, Roopa Mehta, Liliana Muñoz-Hernandez, Ivette Cruz-Bautista, Oscar Perez-Mendez, Maria Teresa Tusie-Luna, Francisco J Gomez-Perez, Päivi Pajukanta, Niina Matikainen, Marja-Riitta Taskinen, Carlos A Aguilar-Salinas

**Affiliations:** Department of Endocrinology and Metabolism, Instituto Nacional de Ciencias Medicas y Nutricion Salvador Zubiran, Mexico City, Mexico; Department of Molecular Biology, Instituto Nacional de Cardiologia Ignacio A. Chavez, Mexico City, Mexico; Molecular Biology and Genomic Medicine Units. Instituto Nacional de Ciencias Medicas y Nutricion Salvador Zubiran. Biomedical Investigation Institute, Universidad Nacional Autonoma de Mexico, Mexico City, Mexico; Department of Human Genetics, David Geffen School of Medicine at UCLA, Los Angeles, CA USA; Diabetes and Obesity Units, Heart and Lung Center, Helsinki University Central Hospital, University of Helsinki, Helsinki, Finland

**Keywords:** Postprandial lipemia, Triglycerides, Apo B-48, Apo A-V, Abdominal obesity, Waist to hip ratio

## Abstract

**Background:**

Alterations in postprandial metabolism have been described in familial combined hyperlipidemia (FCH); however, their underlying mechanisms are not well characterized. We aimed to identify factors related to the magnitude of postprandial lipemia and apolipoprotein (apo) A-V levels in subjects with FCH.

**Methods:**

FCH cases (n = 99) were studied using a standardized meal test. Abdominal obesity was assessed using the waist to hip ratio (WHR). A linear regression model was performed to investigate the variables associated with the triglycerides incremental area under the curve (iAUC). Independent associations between metabolic variables and apo A-V iAUC were also investigated in a randomly selected subgroup (n = 44). The study sample was classified according to the presence of fasting hypertriglyceridemia (≥150 mg/dL) and abdominal obesity (WHR ≥0.92 in men and ≥0.85 in women) to explore differences in parameters.

**Results:**

The fasting apo B-48 levels (r = 0.404), and the WHR (r = 0.359) were independent factors contributing to the triglycerides iAUC (r^2^ = 0.29, P < 0.001). The triglycerides iAUC was independently associated with the apo A-V iAUC (r^2^ = 0.54, P < 0.01). Patients with both hypertriglyceridemia and abdominal obesity showed the most robust triglycerides and apo A-V postprandial responses.

**Conclusions:**

In patients with FCH the fasting apo B-48 level is the main factor associated with postprandial lipemia. Abdominal obesity also contributes to the magnitude of the postprandial response.

The triglycerides postprandial increment is the principal factor associated with the apo A-V postprandial response.

## Background

Postprandial lipemia refers to the increment of triglycerides rich lipoproteins (TRL) in plasma after a meal. This process is associated with an increase in very low density lipoproteins (VLDL) that are transformed to low density lipoproteins (LDL) and delivered to the arterial wall, a decrease of high-density lipoprotein (HDL) particle number and function, and production of small-dense LDL. This unfavorable lipid profile increases cardiovascular disease (CVD) risk [1]. Postprandial lipemia magnitude is affected by the degree of secretion of TRL and their rate of clearance [2].

Fasting triglycerides concentration represents the main determinant of postprandial lipemia, explaining around 30% of the variance [3, 4]. In selected populations, anthropometric characteristics, insulin, HDL-cholesterol (HDL-c), and apolipoprotein (apo) A-IV, have also been identified as determinants of postprandial lipemia [5, 6].

Recent studies have suggested a role for apo A-V in modulating triglycerides metabolism [7]. Firstly, it promotes a greater interaction between TRL and lipoprotein lipase (LPL), increasing their hydrolysis. Secondly, apo A-V promotes liver triglyceride synthesis [8].

Familial combined hyperlipidemia (FCH) is the most common genetic form of dyslipidemia [9]. It is associated with a 1.7- to 10-fold increased risk for CVD [10]. Disturbances of lipid metabolism in FCH are complex. Dyslipidemia in patients with FCH is characterized by an overproduction and a delayed catabolism of VLDL [11]. Patients with FCH exhibit a high VLDL level and an exaggerated postprandial lipemia. The specific mechanisms contributing to postprandial lipemia in FCH are not well established.

In this study we explored the factors associated with postprandial lipemia in patients with FCH and whether the metabolic phenotype alters the magnitude of this phenomenon. We first analyzed the determinants of postprandial triglycerides incremental area under the curve (iAUC). Subsequently, in a randomly selected subgroup, we investigated factors associated with fasting and postprandial Apo A-V. Finally, we classified participants according to the presence of fasting hypertriglyceridemia (HTG) and increased waist to hip ratio (WHR) in order to evaluate if the metabolic phenotype modulated the magnitude and time-sequence of the triglycerides iAUC.

## Methods

### Study subjects

The study population was selected from an out-patient cohort of families with FCH identified at the Instituto Nacional de Ciencias Medicas y Nutricion in Mexico City. None of the subjects were taking lipid-lowering therapy mainly because they were newly diagnosed. FCH was diagnosed using the following criteria: 1) LDL-cholesterol and/or triglycerides concentrations >160 mg/dL and >150 mg/dL, respectively; 2) at least one first-degree relative with hyperlipidemia with a different lipid phenotype, and 3) a concentration of apo B > the 90th percentile for the Mexican population (>108 mg/dL and >99 mg/dL in men and women, respectively) [12–14]. All study subjects had apo B levels over the 90th percentile for the Mexican population and HTG and/or hypercholesterolemia at diagnosis; however, some of them showed inferior levels at the time of the study due to the already known variability in the lipid profile associated with FCH. None of the subjects had evidence of chronic illnesses or significant organ dysfunction. Subjects were weight-stable (<2 kg change) for at least 6 months before entering the study. We excluded subjects with diabetes mellitus, fasting triglycerides ≥1000 mg/dL, body mass index (BMI) ≥40 kg/m^2^, alcohol consumption >10 g/day in women and >20 g/day in men, or currently taking medications known to affect lipid metabolism. Individuals with other primary or secondary dyslipidemias were also excluded.

The Comite de Etica en Investigacion del Instituto Nacional de Ciencias Medicas y Nutricion Salvador Zubiran approved the protocol and informed consent was obtained from all subjects.

### Experimental procedures

Subjects completed a comprehensive medical evaluation, including a history and physical examination. Anthropometric measurements including height, weight, waist, and hip circumference, systolic and diastolic blood pressures were measured following standardized procedures.

### Meal test

Subjects were asked to attend after a 12-hour fasting period, and a standardized meal was provided. Blood samples were then obtained at 0 (fasting), 3, 4, 6, and 8 hours after meal ingestion [15]. The meal consisted of one quarter pounder with cheese hamburger with 5 grams of mayonnaise, 71 grams of fries, and 250 ml of milk. The energy content of the meal is 919 kcal and the composition 50% (51 g) fat, 32.7% (75 g) carbohydrates, and 17.3% (40 g) protein. After ingestion of the meal subjects were allowed to drink only water.

### Sample analysis

Blood samples were collected in EDTA-containing tubes. Levels of triglycerides, total cholesterol, HDL-cholesterol (HDL-c), glucose, aspartate aminotransferase (AST), alanine aminotransferase (ALT), and gamma glutamyltransferase (GGT) were measured by automated enzymatic assays (Beckman Synchron CX, Brea, CA). LDL-cholesterol (LDL-c) was calculated by the Friedewald formula [16] when the fasting triglycerides levels were <300 mg/dL. Insulin levels were determined utilizing micro-particle enzymatic immunoanalysis (MEIA) (Axym System Abbot, Green Oaks, IL) and apo B levels by kinetic nephelometry (Beckman Immage, Brea, CA). In a randomly selected subgroup, apo A-V and apo B-48 concentrations were measured at the University of Helsinki using ELISA assays (Millipore, MA, USA and Shibayagi Co., Shibukawa, Gunma, Japan, respectively).

### Calculations

The iAUC over the 8-hour period was calculated using the trapezoidal method by subtracting the fasting concentration from the total AUC. To evaluate insulin sensitivity the homeostasis model assessment (HOMA-IR) index and the Matsuda index were calculated [17–19].

### Statistical analysis

Data were examined for normality with the Shapiro-Wilk test. Continuous variables are reported as mean ± SD (standard deviation) or median and [interquartile range (IQR)] as appropriate. Categorical variables are reported as frequencies and percentages. Friedman tests were performed to examine the triglycerides, apo B-48, and apo A-V postprandial responses. Following logarithmic transformation of non-normal distributed variables, Pearson correlations were calculated and stepwise multiple linear regression models were constructed to investigate the independent predictors of the iAUC of triglycerides and apo A-V. In the first model, the dependent variable was the triglycerides iAUC and the independent variables were the fasting apo B-48, fasting triglycerides, time of triglycerides peak, WHR, HOMA-IR, and the apo B. In the second model, the dependent variable was the apo A-V iAUC. This model included the triglycerides iAUC, apo B-48 iAUC, fasting triglycerides, apo B, and HDL-c as independent variables.

The population was then classified into four groups according to the presence or absence of HTG (fasting triglycerides level ≥150 mg/dL and <150 mg/dL, respectively) and abdominal obesity (defined using the median WHR of the population, ≥0.92 in men and ≥0.85 and women). One way ANOVA or Kruskal Wallis tests were performed to examine differences between these groups as appropriate. If significance was achieved (P value <0.05) comparisons between individual groups were performed with independent T test or Mann–Whitney U test as appropriate. Statistical analyses were performed using SPSS software version 19.0 (Armonk, NY). A P value <0.05 (two tailed) was considered statistically significant.

## Results

### Baseline characteristics

Of the 106 subjects recruited, seven were excluded; this was due to an unconfirmed diagnosis of FCH (n = 5), presence of undiagnosed diabetes (n = 1), and an elevated fasting triglycerides concentration (n = 1). The characteristics of the 99 individuals participating in the study are summarized in Table 1. They were 45.4 ± 13.5 years old, with overweight (median BMI of 27.1 [25–29.9] kg/m^2^), and had a mean waist circumference of 89.1 ± 10.6 cm. The baseline characteristics of the randomly selected subgroup (n = 44) used for apo A-V and apo B-48 analysis were similar in comparison with the whole cohort (Table 1).

**Table 1:** **Subjects characteristics**

Parameters	All cohort (n = 99)	Subgroup (n = 44)	P
Gender (male/female)	33/66	13/31	0.471
Age (years)	45.4 ± 13.5	46.0 ± 11.6	0.710
BMI (kg/m^2^)^a^	27.1 [25–29.9]	27.0 [25.2–30.5]	0.742
Waist circumference (cm)	89.1 ± 10.6	88.0 ± 11.0	0.358
WHR	0.88 ± 0.07	0.86 ± 0.07	0.070
Systolic blood pressure (mmHg)	120 [110–125]	120 [110–125]	0.742
Diastolic blood pressure (mmHg)	80 [70–80]	77.5 [70–83]	0.462
Triglycerides (mg/dL)	201.5 [135.5–309]	197.0 [131.0–318.0]	0.555
CholedL)/dL)	219.6 ± 37	212.6 ± 32	0.118
HDL-c (mg/dL)	42 [36–51]	39 [34–45]	0.052
LDL-c (mg/dL)^b^	131.4 ± 30.9	133.4 ± 24.5	0.863
Non HDL-c (mg/dl)	175.7 ± 35.9	174.4 ± 32.1	0.757
Apo B (mg/dL)	115.1 ± 25.6	116.9 ± 27.2	0.556
Apo B-48 (μg/mL)	-	5.3 [3–7.4]	-
Apo A-V (ng/mL)	-	303.3 [268.4–390.3]	-
Glucose (mg/dL)	97 [92–107]	94.0 [88–100]	0.054
Insulin (μU/mL)	9.5 [6.8–12.3]	9.5 [7.0–12.6]	0.932
HOMA-IR	2.3 [1.5–3.2]	2.1 [1.4–3.2]	0.390
Matsuda index	30.5 [20.3–39.7]	32.7 [22.1–49.1]	0.259
AST (IU/L)	26 [23–32]	26 [23–32]	0.914
ALT (IU/L)	24.5 [17–34]	22 [17–34]	0.291
GGT (IU/L)	22 [15–37]	20 [14–34]	0.322

Data expressed as means ± SD or medians [IQR].

^a^ The weight in kilograms divided by the square of the height in meters.

^b^ Estimated in 75 subjects with triglycerides levels <300 mg/dl.

Baseline characteristics of study subjects and of the apo A-V and apo B-48 subgroup.

### Postprandial parameters

**Figure 1:**
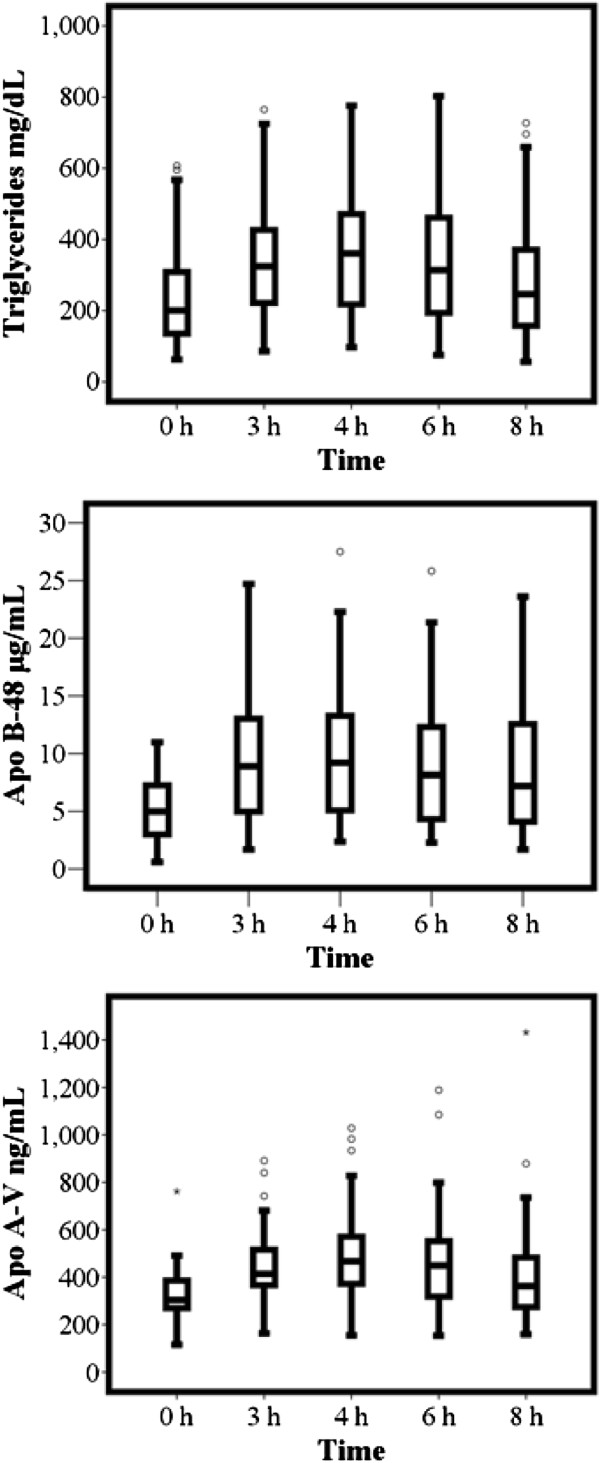
**Triglycerides, apo B-48, and apo A-V levels during the meal test.** Box plots showing median levels of triglycerides, apo B-48, and apo A-V concentrations after the meal test. Boxes show interquartile ranges, and bars represent highest and lowest values (P < 0.001 for all with Friedman test).

After the meal challenge, plasma triglycerides, apo B-48, and apo A-V concentrations increased significantly (P < 0.001) (Figure 1). Median peak level of triglycerides was 367 mg/dL [241.5–510.5]; this was observed at 4 hours in 30.3%, and at 3 hours in 25.3% of the population. At the end of the sampling period, triglycerides concentrations returned to baseline only in 25% of the study subjects. On the other hand, the median apo B-48 peak concentration was 11.7 μg/mL [5.5- 16.3] and the time to peak was at 4 hours in 33%, and at 6 hours in 26% of the population. In the majority of patients (83%) levels remained above baseline after eight hours. In addition, the median apo A-V peak concentration was 478.4 ng/dL [378.6–611.7]. This was observed at 4 hours in 42%, and at 6 hours in 28% of cases. In 67%, concentrations did not reach baseline levels after 8 hours. These results are summarized in Figure 1.

We calculated the apo B48/apo B index, at baseline and at eight hours following the test meal, as a measure of the contribution of chylomicron particles to postprandial lipemia. This index showed a significant change (0.04 [0.03–0.06] vs. 0.06 [0.03–0.11], P < 0.001), representing the chylomicrons increase in response to the meal ingestion.

The fasting and 8-hour apo A-V/triglycerides ratio was estimated to evaluate the change in this index associated with the chylomicrons response after ingestion of the meal. We did not find a significant change in this index comparing the fasting state and 8 hours after ingestion of the meal (1.6 [1.0–2.2] and 1.5 [1.1–2.1], respectively, P = 0.080).

### Factors associated with postprandial lipemia

The triglycerides iAUC showed a significant correlation with the fasting apo B-48 and the fasting triglycerides concentrations (r = 0.43, P = 0.004 and r = 0.39, P < 0.001, respectively). In addition, significant positive correlations were found between the triglycerides iAUC and the time of triglycerides peak (r = 0.35, P < 0.001), WHR (r = 0.30, P = 0.003), apo B (r = 0.24, P = 0.017), BMI (r = 0.24, P = 0.017), and HOMA-IR (r = 0.23, P = 0.022). A significant negative correlation was identified between Matsuda index and the triglycerides iAUC (r = -0.20, P = 0.045).

Despite a positive correlation between the triglycerides iAUC and the apo B-48 iAUC (r = 0.44, P = 0.004), we did not find significant correlations between the apo B-48 iAUC and the studied variables.

### Independent factors associated with post-prandial triglycerides response

The linear regression analysis identified the fasting apo B-48 levels (r = 0.40), and the WHR (r = 0.35) as independent parameters determining the triglycerides iAUC, explaining 29% of the variability. The model also included the fasting triglycerides concentration, time of triglycerides peak, HOMA-IR, and the apo B levels, none of these parameters demonstrated an independent association with the triglycerides iAUC (Table 2).

**Table 2:** **Linear regression model**

Independent variables	β	Standardized β	t	P	Partial correlations
Fasting apo B-48	0.512	0.377	2.45	0.009	0.404
WHR	4.885	0.328	2.4	0.021	0.359

r^2^ = 0.29, F = 7.97, P < 0.001.

Dependent variable: triglycerides iAUC; variables included in the model: fasting apo B-48, fasting triglycerides, time of triglycerides peak, apolipoprotein B, HOMA-IR, and waist to hip ratio (WHR).

Stepwise linear regression analysis showing independent variables associated with postprandial triglycerides levels in FCH.

### Factors associated with fasting and postprandial apo A-V levels

We explored associations between the fasting apo A-V concentration, apo A-V iAUC, and anthropometric and metabolic parameters. The fasting apo A-V concentration showed significant correlations with the fasting triglycerides levels (r = 0.39, P = 0.010) and apo B levels (r = 0.32, P = 0.032).

The apo A-V iAUC showed significant associations with the triglycerides iAUC (r = 0.72, P < 0.001), apo B levels (r = 0.52, P = 0.001), apo B-48 iAUC (r = 0.47, P = 0.004), and fasting triglycerides levels (r = 0.46, P = 0.004). A negative correlation between the apo A-V iAUC and HDL-c was also found (r = -0.36, P = 0.028).

### Independent factors associated with postprandial apo A-V response

In the linear regression model, the only variable independently associated with the apo A-V iAUC was the triglycerides iAUC, explaining 54.4% of the variability in this variable (P < 0.001). This model also included the apo B-48 iAUC, fasting triglycerides, apo B, and HDL-c levels as independent variables. When the triglycerides iAUC was removed from this model, the apo B-48 iAUC, apo B, and HDL-c were associated with the postprandial apo A-V response, explaining 51.1% of the variability in this parameter.

### Parameters according to the presence or absence of fasting hypertriglyceridemia and abdominal obesity

Individuals were classified in four groups according to the level of fasting triglycerides and the median WHR as follows: 1) HTG (≥150 mg/dL) with abdominal obesity (WHR ≥0.92 in men and ≥0.85 in women), 2) HTG without abdominal obesity, 3) normotriglyceridemia (NTG) (<150 mg/dl) with abdominal obesity, and 4) NTG without abdominal obesity. The characteristics of these groups are shown in Table 3.

**Table 3:** **Subjects subgroups characteristics**

Variable	HTG with obesity (n = 48)	HTG without obesity (n = 21)	NTG with obesity (n = 13)	NTG without obesity (n = 17)	P
WHR	0.92 ± 0.05^c, e^	0.82 ± 0.04^d^	0.91 ± 0.04^e^	0.80 ± 0.03	<0.001
**Fasting parameters** ^**a**^					
Triglycerides (mg/dL)	275 [201–345]^d, e^	271 [185–346]^d, e^	126 [103–136]	105 [79–131]	<0.001
Cholesterol (mg/dL)	226.9 ± 37.2^d, e^	228.8 ± 38.9	206.3 ± 18.9	197.5 ± 34.9	0.010
LDL-c (mg/dL)	129.4 ± 26.4	141.9 ± 44.7	133.7 ± 16.7	129.3 ± 24.9	0.604
Non HDL-c (mg/dL)	173.3 ± 27.2	181.6 ± 41.7^d, e^	157.7 ± 16.1	150 ± 29.4	0.014
Apo B (mg/dL)	123.2 ± 24.9^d, e^	122.3 ± 23.1	109.1 ± 11.8	107.3 ± 24.2	<0.001
Apo B-48 (μg/mL)^b^	6.5 [5.4–8.9]^d, e^	6.7 [3–7.4]^d^	1.5 [0.9–2.9]	3.1 [1.3–4.6]	<0.001
Apo A-V (ng/mL)^b^	365.5	303.3	215.5	287.9	0.096
	[301.7–408.3]	[268.4–374.3]	[187.5–326.2]	[267.5–329.8]	
Glucose (mg/dL)	103 [95–110]^d, e^	97 [92–107]	96 [91–101]	91 [87–94]	0.001
Insulin (μU/mL)	11.15 [7.7–13.4]^d, e^	8.6 [5.6–12.6]	7.6 [6.1–9.5]	8.9 [5.1–9.8]	0.029
HOMA-IR	2.73 [1.91–3.61]^d, e^	2.03 [1.45–3.21]	1.89 [1.41–2.5]	2.04 [1.28–2.29]	0.010
AST (IU/L)	30 [25–33]^d^	26 [24–33]	25 [21.5–28]	25 [22–27]	0.073
ALT (IU/L)	30 [21–37]^d, e^	22 [18–34]	19.5 [15–25]	20 [16–30]	0.015
GGT (IU/L)	28 [18–38]	19 [15–47]	23.5 [10–34.5]	15 [13–29]	0.085
Apo B-48/apo B^b^	0.056 ± 0.0^d^	0.046 ± 0.02	0.015 ± 0.00	0.041 ± 0.02	0.035
Apo A-V/triglycerides^b^	1.29 [1.02–1.73]^e^	1.10 [0.87–1.54]^e^	1.84 [1.60–2.48]	3.06 [2.24–4.27]	0.001
**Postprandial parameters**					
Triglycerides iAUC	777.2	576.5	433	439.5	<0.001
(mg/dL/h)	[548.5–1092.7]^d, e^	[213–1028]	[280–595.5]	[243.5–568.5]	
Triglycerides peak (mg/dL)	444.5 [353–562]^d, e^	444 [268–570]^d, e^	228 [188–291]	211 [180–231]	<0.001
Triglycerides time to peak (h)	5 [4–6]	4 [4–5]	4 [4–5]	3 [3–4]	0.075
Apo B-48 iAUC (μg/mL/h)^b^	27.1 [15.3–49.3]	23.6 [11.6–29.3]	15 [10.8–23.4]	18.7 [8.7–24.6]	0.373
Apo B-48 peak (μg/mL)^b^	15.8 [11.6–22.2]^d, e^	14 [7–16.7]	4.2 [3.1–7.4]	7.4 [3.9–10]	0.002
Apo B-48 time to peak (h)^b^	6 [4–8]^e^	6 [4–8]^e^	5 [3.5–6]	4 [3–4]	0.024
Apo A-V iAUC	1132.9	707.8	363.8	145.1	0.002
(ng/mL/h)^b^	[826.4–1474.7]^d, e^	[72.6–1533.8]	[145.0–480.5]	[–5.8–453.0]	
Apo A-V peak	603.4	459	323.2	358.6	<0.001
(ng/mL)^b^	[534–673.7]^d, e^	[382.9–613.9]	[226.7–439.3]	[299.2–423.9]	
Apo A-V time to peak (h)^b^	6 [4–6]^e^	4 [4–6]	5 [3.5–6]	3 [3–4]	0.006

Data are expressed as means ± SD or medians [IQR]. HTG: hypertriglyceridemia defined as fasting triglycerides ≥150 mg/dL. Obesity defined as waist to hip ratio (WHR) ≥0.92 in males and ≥0.85 in females. NTG: normotriglyceridemia defined as fasting triglycerides <150 mg/dL. ^a^All study subjects had apo B levels over the 90th percentile for the Mexican population and HTG and/or hypercholesterolemia at diagnosis; however, some of them showed inferior levels at the time of the study due to the already known variability in the lipid profile associated with FCH.

^b^N = 44. ^c^Significantly different from individuals with HTG without obesity. ^d^Significantly different from individuals with NTG with obesity. ^e^Significantly different from individuals with NTG without abdominal obesity.

Subjects characteristics according to the presence of hypertriglyceridemia and abdominal obesity.

Individuals with fasting HTG and abdominal obesity, showed the highest triglycerides iAUC, and the time to triglycerides peak concentration was later (median 5 hours) than in the other groups. In this group apo B-48 peak concentrations were also higher. Triglycerides peak level was higher in the groups with fasting HTG in comparison with the groups with normal fasting triglycerides concentration. The apo B-48 iAUC showed a tendency to be higher in groups with fasting hypertriglyceridemia.

**Figure 2:**
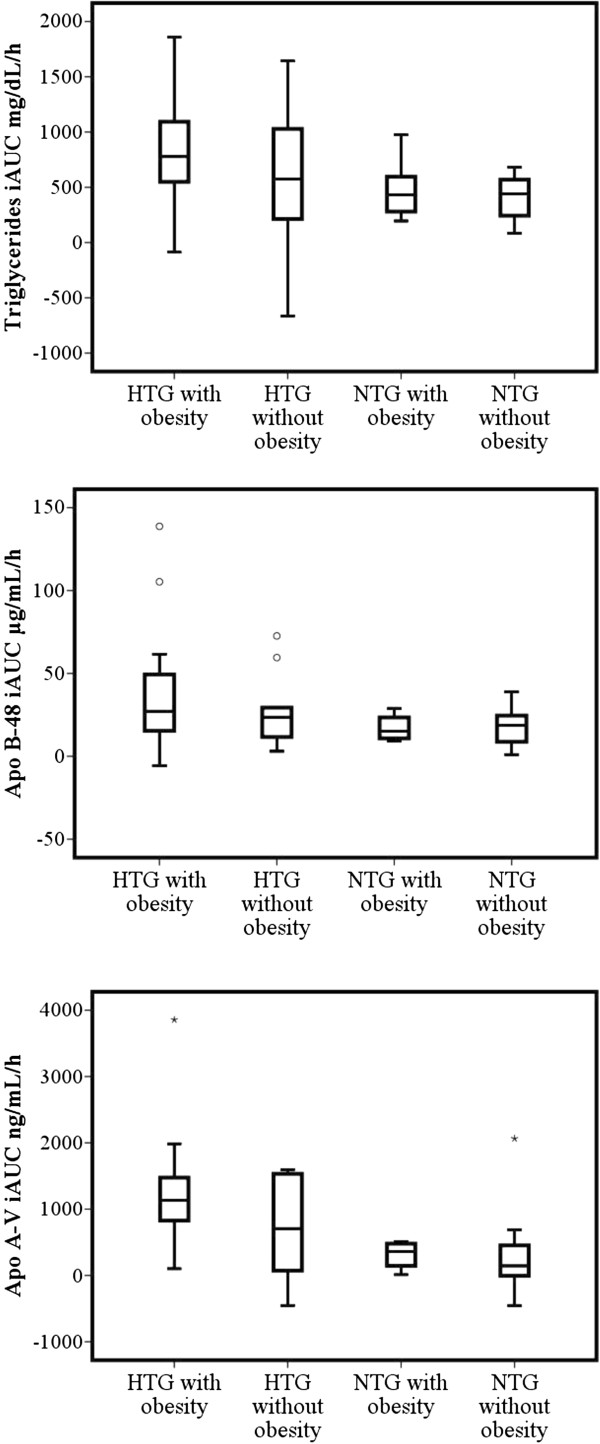
**Triglycerides iAUC, apo B-48 iAUC, and apo A-V iAUC in subgroups according hypertriglyceridemia and abdominal obesity.** Box plots showing median levels of triglycerides iAUC, apo B-48 iAUC, and apo A-V iAUC after the meal test. Boxes show interquartile ranges, and bars represent highest and lowest values P < 0.001 for triglycerides iAUC, P = 0.373 for apo B-48 iAUC, and P = 0.002 for apo A-V iAUC with Kruskal Wallis test.

The highest apo A-V iAUC and peak concentrations were observed in the HTG with abdominal obesity group, followed by the group with HTG without abdominal obesity. The group without any of the abnormalities showed the shortest time to peak apo A-V levels. In contrast, the group with both HTG and abdominal obesity showed a later peak. These results are summarized in Figure 2 and Table 3.

## Discussion

The lipoproteins and mechanisms that modulate postprandial lipemia in patients with FCH are not well understood. We studied a cohort of patients with FCH to determine factors related to postprandial lipemia. Our results show that the magnitude of the triglycerides iAUC is determined by the fasting apo B-48 concentration and the WHR. In our second linear regression model the postprandial apo A-V levels were only associated with the triglycerides iAUC. When the study population was classified according to the fasting triglycerides level and the WHR, the most robust postprandial triglycerides response was observed in the group with fasting HTG and abdominal obesity.

These results indicate that the magnitude of the postprandial lipemia in patients with FCH is determined by the fasting apo B-48 concentration and potentiated by the presence of abdominal obesity. However, the linear regression model explained only a modest proportion of the variability in postprandial lipemia; therefore, additional variables related to this phenomenon may be involved.

Interestingly, the apo B-48 postprandial response, reflecting the chylomicrons rise after the meal, was not independently associated with abdominal obesity or fasting hypertriglyceridemia. This result indicates that in patients with FCH the chylomicron response is not determined by the same parameters that influence the postprandial triglycerides iAUC (fasting apo B-48 levels and abdominal obesity).

In contrast to our results, other studies including individuals with diverse cardiovascular risk have reported that fasting triglycerides levels is the major determinant of postprandial lipemia [3, 5, 6, 20]. The novel finding of an independent association between fasting apo B-48 levels and postprandial lipemia in FCH indicate a potential role for intestinal-derived lipoproteins in postprandial metabolism in individuals with FCH.

The relationship between abdominal obesity and postprandial lipemic response is less clear. Abdominal adiposity may be associated with liver fat deposition, a driving force for the overproduction of VLDL [11]. We found a strong and independent association between the WHR, a validated marker of abdominal obesity, and postprandial lipemia. This result is consistent with the finding that patients with fasting HTG and abdominal obesity showed the greatest postprandial triglycerides levels. Studies have confirmed that abdominal obesity, a marker of visceral fat accumulation, is independently associated with the magnitude of postprandial lipemia [5, 6, 21, 22]. These conclusions are in agreement with our findings.

In patients with FCH the postprandial lipid response was aggravated by abdominal obesity. A feasible mechanism for explaining our results is the oversaturation and competition of chylomicrons and VLDL particles for the same removal pathways [23]. This condition added to other known cardiovascular risk factors may play a key role in the increased risk for cardiovascular disease seen in these individuals [1].

A novel finding of our report is the analysis of the fasting and postprandial apo A-V response. Apo A-V postprandial levels were mainly associated with the postprandial triglycerides iAUC. Apo A-V is produced by the liver and is a component of VLDL and HDL lipoprotein fractions [24]. It is presumed to be a factor in the activation of LPL resulting in increased triglycerides hydrolysis [8]. In individuals with type 2 diabetes following a fat load, the apo A-V postprandial response paralleled the increase in VLDL-triglycerides and apo C-III levels. However, an association between the apo A-V postprandial levels and LPL activity was not found [25]. In FCH, polymorphisms on the *APOAV* have been associated with increased fasting triglycerides concentrations [26]. In this study, following the fat meal, apo A-V levels increased and did not return to the baseline at the end of the test. Interestingly, we found a significant correlation between the apo B-48 and apo A-V postprandial responses. We consider that this finding could reflect an association of apo A-V not only with the VLDL particles, but also with chylomicrons [27]. In the linear regression analysis the only variable independently associated with the postprandial apo A-V response was the triglycerides iAUC. Moreover, after analyzing the subgroups according to their fasting triglyceride levels, the groups with fasting HTG showed a significantly higher postprandial apo A-V response in comparison to the normal fasting triglycerides levels groups. These results confirm that apo A-V is associated with the TRL in the postprandial period, and is in line with the known regulation of VLDL metabolism by apo A-V [8, 26].

Our study has limitations. The cross-sectional design only suggests associations and not causality. In addition, we did not use DEXA or imaging studies to measure fat mass and visceral adipose tissue volume. This would have allowed more precise quantification of abdominal obesity; however, the WHR is a validated marker of visceral tissue accumulation [28]. Also, the WHR is an informative and simple measure to perform in everyday clinical setting. Apo A-V polymorphisms were not investigated in this study. Apo A-V was analyzed in a randomly selected subgroup of the population; nevertheless, this subgroup was representative of the complete sample studied. Finally, we did not include a control group without FCH to compare our results because our focus was to identify the variables related with postprandial lipemia in FCH patients.

## Conclusions

In conclusion, in individuals with FCH fasting apo B-48 concentration is the main factor associated with the magnitude of postprandial lipemia. This response is potentiated by abdominal obesity. Finally, the Apo A-V postprandial levels are associated with the postprandial triglycerides increment.

## Abbreviations

FCH: Familial combined hyperlipidemia
iAUC: Incremental area under the curve
WHR: Waist to hip ratio
TRL: Triglycerides rich lipoproteins
CVD: Cardiovascular disease
VLDL: Very low density lipoproteins
HDL: High density lipoprotein
HDL-c: HDL-cholesterol
LPL: Lipoprotein lipase
BMI: Body mass index
AST: Aspartate aminotransferase
ALT: Alanine aminotransferase
GGT: Gamma glutamyltransferase
LDL-c: LDL-cholesterol
MEIA: Micro-particle enzymatic immunoanalysis
ELISA: Enzyme-linked immunosorbent assays
SD: Standard deviation
IQR: Interquartile range
ANOVA: Analysis of variance
HTG: Hypertriglyceridemia
NTG: Normotriglyceridemia.

## References

[1] Goldberg IJ, Eckel RH, McPherson R (2011). Triglycerides and heart disease: still a hypothesis?. Arterioscler Thromb Vasc Biol.

[2] Cohn JS, Johnson EJ, Millar JS, Cohn SD, Milne RW, Marcel YL, Russell RM, Schaefer EJ (1993). Contribution of apoB-48 and apoB-100 triglyceride-rich lipoproteins (TRL) to postprandial increases in the plasma concentration of TRL triglycerides and retinyl esters. J Lipid Res.

[3] Hwu CM, Lin MW, Liou TL, Hsiao LC, Liang KW, Tsai TT, Ho LT (2008). Fasting triglyceride is a major determinant of postprandial triglyceride response in postmenopausal women. Menopause.

[4] Tan KC, Tso AW, Ma OC, Pang RW, Tam S, Lam KS (2005). Determinants of postprandial triglyceride and remnant-like lipoproteins in type 2 diabetes. Diabetes Metab Res Rev.

[5] van Wijk JP, Halkes CJ, Erkelens DW, Castro Cabezas M (2003). Fasting and daylong triglycerides in obesity with and without type 2 diabetes. Metab Clin Exp.

[6] Pirro M, Lupattelli G, Siepi D, Palumbo B, Roscini AR, Marchesi S, Schillaci G, Mannarino E (2001). Postprandial lipemia and associated metabolic disturbances in healthy and hyperlipemic postmenopausal women. Metab Clin Exp.

[7] Sharma V, Forte TM, Ryan RO (2013). Influence of apolipoprotein A-V on the metabolic fate of triacylglycerol. Curr Opin Lipidol.

[8] van Dijk KW, Rensen PC, Voshol PJ, Havekes LM (2004). The role and mode of action of apolipoproteins CIII and AV: synergistic actors in triglyceride metabolism?. Curr Opin Lipidol.

[9] Aguilar Salinas CA, Zamora M, Gomez-Diaz RA, Mehta R, Gomez Perez FJ, Rull JA (2004). Familial combined hyperlipidemia: controversial aspects of its diagnosis and pathogenesis. Semin Vasc Med.

[10] Carr MC, Brunzell JD (2004). Abdominal obesity and dyslipidemia in the metabolic syndrome: importance of type 2 diabetes and familial combined hyperlipidemia in coronary artery disease risk. J Clin Endocrinol Metab.

[11] Castro Cabezas M (2003). Postprandial lipaemia in familial combined hyperlipidaemia. Biochem Soc Trans.

[12] Valles V, Aguilar-Salinas CA, Gomez-Perez FJ, Rojas R, Franco A, Olaiz G, Rull JA, Sepulveda J (2002). Apolipoprotein B and A-I distribution in Mexican urban adults: results of a nationwide survey. Metab Clin Exp.

[13] del Rincon Jarero JP, Aguilar-Salinas CA, Guillen-Pineda LE, Gomez-Perez FJ, Rull JA (2002). Lack of agreement between the plasma lipid-based criteria and apoprotein B for the diagnosis of familial combined hyperlipidemia in members of familial combined hyperlipidemia kindreds. Metab Clin Exp.

[14] Mata P, Alonso R, Ruiz Garcia A, Diaz Diaz JL, Gonzalez N, Gijon Conde T, Martinez Faedo C, Moron I, Arranz E, Aguado R, Argueso R, Perez de Isla L (2014). Familial combined hyperlipidemia: consensus document. Atencion Primaria Sociedad Espanola Medicina Familia y Comunitaria.

[15] Syvanne M, Talmud PJ, Humphries SE, Fisher RM, Rosseneu M, Hilden H, Taskinen MR (1997). Determinants of postprandial lipemia in men with coronary artery disease and low levels of HDL cholesterol. J Lipid Res.

[16] Friedewald WT, Levy RI, Fredrickson DS (1972). Estimation of the concentration of low-density lipoprotein cholesterol in plasma, without use of the preparative ultracentrifuge. Clin Chem.

[17] Matthews DR, Hosker JP, Rudenski AS, Naylor BA, Treacher DF, Turner RC (1985). Homeostasis model assessment: insulin resistance and beta-cell function from fasting plasma glucose and insulin concentrations in man. Diabetologia.

[18] Matsuda M, DeFronzo RA (1999). Insulin sensitivity indices obtained from oral glucose tolerance testing: comparison with the euglycemic insulin clamp. Diabetes Care.

[19] Maki KC, Kelley KM, Lawless AL, Hubacher RL, Schild AL, Dicklin MR, Rains TM (2011). Validation of insulin sensitivity and secretion indices derived from the liquid meal tolerance test. Diabetes Technol Ther.

[20] Kolovou GD, Anagnostopoulou KK, Pavlidis AN, Salpea KD, Iraklianou SA, Tsarpalis K, Damaskos DS, Manolis A, Cokkinos DV (2005). Postprandial lipemia in men with metabolic syndrome, hypertensives and healthy subjects. Lipids Health Dis.

[21] Sharrett AR, Heiss G, Chambless LE, Boerwinkle E, Coady SA, Folsom AR, Patsch W (2001). Metabolic and lifestyle determinants of postprandial lipemia differ from those of fasting triglycerides: the Atherosclerosis Risk In Communities (ARIC) study. Arterioscler Thromb Vasc Biol.

[22] Guerci B, Verges B, Durlach V, Hadjadj S, Drouin P, Paul JL (2000). Relationship between altered postprandial lipemia and insulin resistance in normolipidemic and normoglucose tolerant obese patients. Int J Obes Relat Metab Disord.

[23] Meijssen S, Cabezas MC, Twickler TB, Jansen H, Erkelens DW (2000). In vivo evidence of defective postprandial and postabsorptive free fatty acid metabolism in familial combined hyperlipidemia. J Lipid Res.

[24] Pennacchio LA, Olivier M, Hubacek JA, Cohen JC, Cox DR, Fruchart JC, Krauss RM, Rubin EM (2001). An apolipoprotein influencing triglycerides in humans and mice revealed by comparative sequencing. Science.

[25] Kahri J, Fruchart-Najib J, Matikainen N, Fruchart JC, Vakkilainen J, Taskinen MR (2007). The increase of apolipoprotein A-V during postprandial lipemia parallels the response of triglyceride-rich lipoproteins in type 2 diabetes: no relationship between apoA-V and postheparin plasma lipolytic activity. Diabetes Care.

[26] Ribalta J, Figuera L, Fernandez-Ballart J, Vilella E, Castro Cabezas M, Masana L, Joven J (2002). Newly identified apolipoprotein AV gene predisposes to high plasma triglycerides in familial combined hyperlipidemia. Clin Chem.

[27] O'Brien PJ, Alborn WE, Sloan JH, Ulmer M, Boodhoo A, Knierman MD, Schultze AE, Konrad RJ (2005). The novel apolipoprotein A5 is present in human serum, is associated with VLDL, HDL, and chylomicrons, and circulates at very low concentrations compared with other apolipoproteins. Clin Chem.

[28] Welborn TA, Dhaliwal SS (2007). Preferred clinical measures of central obesity for predicting mortality. Eur J Clin Nutr.

[CR29] The pre-publication history for this paper can be accessed here: http://www.biomedcentral.com/1472-6823/14/90/prepub

